# Cesarean Audit Using Robson Classification at a Tertiary Care Center in Bihar: A Retrospective Study

**DOI:** 10.7759/cureus.23133

**Published:** 2022-03-13

**Authors:** Pammy Pravina, Ranjana Ranjana, Neeru Goel

**Affiliations:** 1 Obstetrics and Gynecology, Indira Gandhi Institute of Medical Sciences, Patna, IND

**Keywords:** robson’s ten group classification system, indications of cesarean section, cesarean section rate, cesarean section, audit

## Abstract

Background

Since 1985, the international healthcare community has considered the ideal rate for cesarean section (CS) to be between 10% and 15%.However, CS has been increasing both in developed and developing countries. The aim of the present study was to audit CS using Robson’s Ten Group Classification System (TGCS).

Methodology

This retrospective, hospital record-based study was conducted over a period of three years from April 1, 2016, to March 31, 2019, in the Department of Obstetrics and Gynecology at Indira Gandhi Institute of Medical Sciences, Patna, Bihar, India. Data of patients who delivered by CS during this period were recorded and categorized in the 10 groups of TGCS. The size of each group, CS rate, and contribution of each group toward overall CS were calculated. Indications of CS in each group were analyzed, and strategies were planned to optimize the use of CS. The Chi-square test was used to analyse the statistical significance of the differences in the number of CS between the different Robson's groups.

Results

The total number of deliveries was 2,128 during the study period, of which CS was performed in 812 deliveries, with a CS rate of 38.16% in our institute. Robson’s group 5 (34.97%) was the major contributor to the overall CS rate, followed by group 2 (26.35%), group 1 (15.51%), and group 10 (7.14%). The incidence of primary CS (61.82%) was more than repeat CS (38.17%). Previous CS, fetal distress, failed induction, arrest of labor, and malpresentation were the main indications for CS.

Conclusions

Robson’s TGCS serves as an important tool for auditing CS. Indications of CS among major contributors and primary group should be analyzed regularly and uniform and standard protocols should be used. Standardization of indications for CS, regular audits, and definite protocols will help in reducing the CS rate in our hospital.

## Introduction

Since 1985, the international healthcare community has considered the ideal rate for cesarean section (CS) to be between 10% and 15% [1]. However, the incidence of CS has been increasing both in developed and developing countries. According to the latest data (National Family Health Survey 2015-2016, NFHS-4), the CS rate at population level in India appears to be 17.2%, and globally it is 21% of all births [2].

Although CS is a lifesaving procedure for the fetus, the mother, or both in certain cases, it should be done under ideal conditions with a valid obstetrical indication. Unindicated CS without evidence of concomitant decrease in maternal or neonatal morbidity and mortality should be avoided to minimize its implications in the index or future pregnancies, as well as to reduce the burden of cost on the healthcare system.

Immediate and long-term complications of CS include increased risk of maternal morbidity and mortality, postpartum hemorrhage, increased need for blood transfusion, longer hospitalization, postpartum infections, and retained and adherent placenta [3,4].

Strategies to reduce CS while maintaining safe outcomes for both the mother and the infant require continuous auditing of CS, implementation of effective strategies to optimize CS rates, and improvement in clinical practices and quality care to patients. The World Health Organization (WHO) in 2015 and the International Federation of Gynecology and Obstetrics (FIGO) in 2016 proposed the use of the Robson Classification (also known as the Ten Group Classification) as a global standard for assessing, monitoring, and comparing CS rates both within healthcare facilities, over time, and between facilities [1,5,6]. This system classifies all women into one of 10 categories that are mutually exclusive and, as a set, completely comprehensive. The categories are based on five basic obstetrical characteristics (parity, number of fetuses, previous CS, onset of labor, gestational age, and fetal presentation).

Kacerauskiene et al. [7] employed the Robson Classification in 19 Lithuanian hospitals. There was overall reduction in CS rate from 26.9% in 2012 to 22.7% in 2014 (p < 0.001). Similarly, in the study conducted by Ansari et al. [8], re-audit of cesarean deliveries showed a significant reduction in CS among major contributors, as well as a reduction in overall CS rate from 54% to 38.2%.

To date, no study has audited CS rates in Bihar using Robson criteria. Therefore, in this study, we attempt to classify CS based on this system to report CS rate in our scenario, as well as to determine which clinically relevant groups contributed most to the CS in our institution.

The aim of the present study was to audit CS using Robson’s Ten Group Classification System (TGCS). The primary objective of this study was to analyze the prevalence rate of CS in our scenario using TGCS and to identify the main contributors of each subgroup to the overall CS rate to plan further interventions. Comparison of our rate of CS in each group with the standard and national data and maternal and perinatal outcomes were considered secondary objectives.

## Materials and methods

Study design and participants

This retrospective, hospital record-based study was conducted over a period of three years from April 1, 2016, to March 31, 2019, in the Department of Obstetrics and Gynecology at Indira Gandhi Institute of Medical Sciences (IGIMS), Patna, Bihar. Ethical clearance was obtained for the study from the Institutional Ethical Committee of our Institute (Letter No.: 843/IEC/IGIMS/2019, dated 09/04/2019).

Sample size calculation

A total of 812 pregnant women delivered by CS during the study period. In the study by Abubeker et al., the CS rate was 34.7% [9] The largest contributors to the overall CS rate were group 10 (19.1%), group 2 (18.3%), group 5 (17.1%), and group 4 (15.8%). Taking this value as reference, the minimum required sample size with a 3.5% margin of error and a 5% level of significance was 710 patients. To reduce the margin of error, the total sample size in this study was 812. The following formula was used to calculate sample size: N ≥ (p(1 -p))/(ME/zα)2, where Zα is the value of Z at a two-sided alpha error of 5%, ME is the margin of error, and p is the CS rate or contributors to the overall CS rate.

Inclusion and exclusion criteria

**Figure 1:**
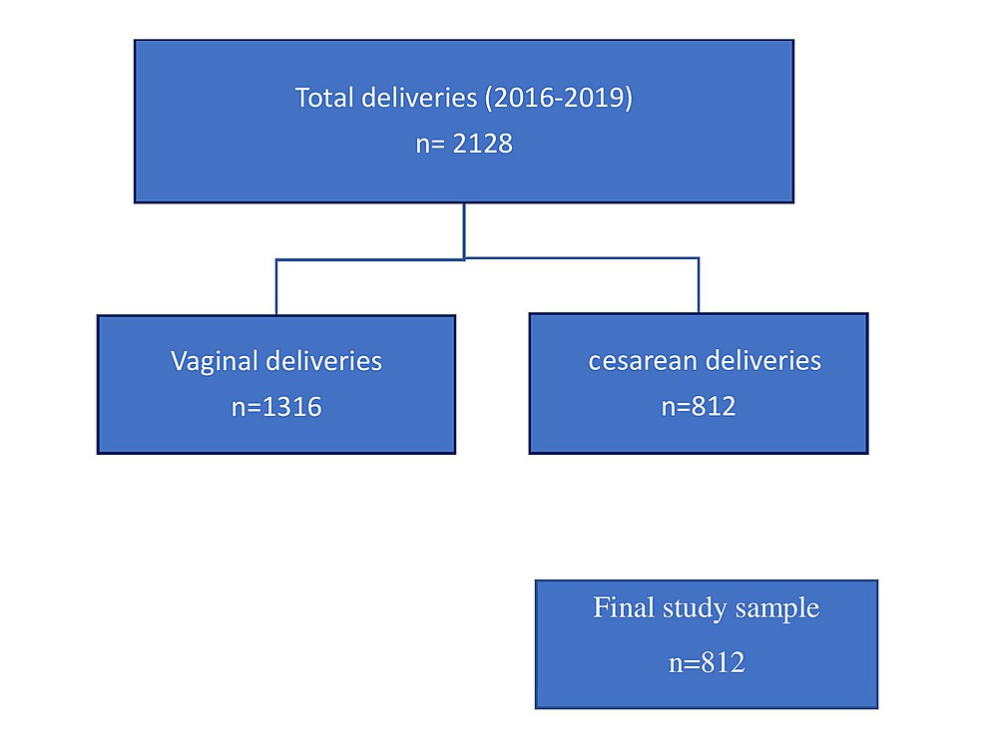
Flowchart of deliveries in our study population.

Patients who delivered by CS during the given period were included in this study. Term and preterm normal or instrumental vaginally delivered patients were excluded from this study. Figure 1 presents a flowchart of deliveries in our study population.

Study setting

IGIMS, Sheikhpura, Patna, Bihar is a tertiary care hospital with a superspeciality department and is the main referral center for complicated cases (pregnancy with complicated heart diseases, hepatic and renal diseases, in addition to all other high-risk pregnancies) from private, primary, and secondary health institutions in Bihar state. In our institute, around 1,000 deliveries occur annually.

Study procedure

In this study, 812 cesarean deliveries during the three-year study period were categorized in the 10 groups of TGCS, as shown in Table 1. Relevant information regarding each CS case was obtained from the hospital records of the medical record section, and the total number of deliveries was matched with the labor room record. A proforma was designed and details of each CS case was entered (age, parity, mode of previous deliveries, gestational age, number of fetuses, fetal presentation, previous CS and its indications, the onset of labor; fetal outcomes: birth weight, APGAR score, and fetal complications; maternal complications: postpartum hemorrhage, anemia, wound infection, need for blood transfusion, ruptured uterus, intensive care unit (ICU) admission, and maternal mortality).

**Table 1:** Robson’s Ten Group Classification System. CS: cesarean section

Group 1	Nulliparous, single, cephalic pregnancy >37 weeks in spontaneous labor
Group 2	Nulliparous, single, cephalic pregnancy >37 weeks who had labor induced or delivered before labor by CS
Group 3	Multiparous, without previous uterine scar with single, cephalic pregnancy >37 weeks in spontaneous labor
Group 4	Multiparous, without previous uterine scar with single, cephalic pregnancy >37 weeks who had labor induced or delivered before labor by CS
Group 5	All multiparous with at least one previous uterine scar, with single cephalic pregnancy >37 weeks
Group 6	All nulliparous with a single breech pregnancy
Group 7	All multiparous with a single breech including women with previous scars
Group 8	All women with multiple pregnancies including those with uterine scars
Group 9	All women with a single pregnancy with transverse or oblique lie including women with previous scars
Group 10	All women with single, cephalic <37 weeks including women with previous scars

Statistical analysis

Data of individual cases were entered in a Microsoft Excel spreadsheet, and the final analysis was done using SPSS software, version 21 (IBM Corp., Armonk, NY, USA). Categorical variables were presented as numbers and percentages (%). On the other hand, quantitative data were presented as the means ± standard deviation (SD). The chi-square test was used to analyze the statistical significance of the differences in the numbers of CS between the different Robson groups. The size of each Robson group, CS rate, and the contribution of each group toward overall CS were calculated. Indications of CS in each group were analyzed, and strategies were planned to optimize the use of CS.

## Results

Of the 2,128 deliveries during the study period, the number of CS was 812, with a CS rate of 38.16% in our institute. Out of these, elective CS was 39.06% and emergency CS was 60.93%.

The majority of the study participants were in the age group of 20-35 years (96.79%). Parity between one and two was seen in 45.81% of women, while 44.08% of women were nulliparous, and multiparous women constituted only 10.09% of the study population. Among the study participants, a history of previous CS was present in 36.94% of women, while 63.05% of women had an unscarred uterus. Most of the CS was at term gestational age (66.25%). Post-dated CS was noted in 14.28% of women, while preterm CS was noted in 19.45% of women (Table 2). Among the study participants, 45.07% of patients were admitted to the labor room with labor pain. Induction of labor was done in 32.51% of cases, while 22.41% of patients were taken directly for CS without prior labor pain (Table 2). The cephalic presentation was the most common fetal presentation seen in 89.40% of cases and 98.52% of fetuses were singleton. Fetal outcomes are presented in Table 2.

**Table 2:** Sociodemographic and obstetric conditions of the study participants. CS: cesarean section; NICU: neonatal intensive care unit; PPH: postpartum hemorrhage; AKI: acute kidney injury; ICU: intensive care unit

Variables	Number (n)	Percentage (%)
Age (years)		
<20	16	1.97%
20–35	786	96.79%
>35	10	1.23%
Parity		
Nulliparous	358	44.08%
1-2	372	45.81%
>2	82	10.09%
Previous CS		
No	512	63.05%
Yes	300	36.94%
Gestational age at delivery		
<37 weeks (preterm)	158	19.46%
37–40 weeks (term)	538	66.26%
>40 weeks (post-dated)	116	14.28%
Onset of labor		
Spontaneous	366	45.07%
Induced	264	32.51%
No labor (prelabor CS)	182	22.41%
Fetal presentation		
Cephalic	726	89.40%
Breech	68	8.37%
Transverse/oblique/brow/others	18	2.21%
Number of fetuses		
Singleton	800	98.52%
Multiple	12	1.47%
Fetal status at birth		
Alive	818	99.27%
Stillbirths	3	0.36%
Intrauterine death	3	0.36%
Apgar score at five minutes		
<7	34	4.12%
>7	790	95.87%
Birth weight (g)		
<1,500	8	0.97%
1,500–2,499	86	10.43%
2,500–3,999	696	84.46%
≥4,000	34	4.12%
NICU admission	67	8.13%
Neonatal mortality	9	1.09%
Maternal morbidity and mortality		
PPH	52	6.4%
Moderate/Severe Anemia	192	23.64%
Wound infection	16	1.97%
Postpartum AKI	4	0.49%
Blood transfusion	110	13.54%
Rupture uterus	8	0.98%
ICU admission	34	4.18%
Maternal mortality	10	1.23%

In our study, maternal complications were seen in 52.46% of the study population. These complications included postpartum hemorrhage (6.4%), moderate/severe anemia (23.64%), wound infection (1.97%), postpartum acute kidney injury (0.49%), blood transfusion (13.54%), ruptured uterus (0.98%), ICU admission (4.18%), and maternal mortality (1.23%) (Table 2).

Robson’s TGCS

In our study, group 5 (multiparous with prior CS, singleton, cephalic, ≥37 weeks) were the highest contributors to the overall CS rate, contributing 34.97% of all CS and 13.34% to all deliveries. Group 2 (nulliparous, singleton, cephalic, ≥37 weeks, induced labor or CS before labor) were the second highest contributors, contributing 26.35% to the overall CS and 10.05% to all deliveries. The third highest contributors were single cephalic nulliparous women at term and in spontaneous labor (group 1) contributing 15.51% to the overall CS rate and 5.92% of all deliveries. The fourth highest contributors were singleton, cephalic, ≤36 weeks, including previous CS (group 10) contributing 7.14% to the overall CS rate and 2.72% of all deliveries. The remaining groups (groups 3, 4, 6, 7, 8, and 9) contributed 16% of all CS and 6.11% of total deliveries (Table 3). The Chi-square test showed that the CS rate was significantly higher in groups 5, 2, and 1 compared to other Robson groups (p-value < 0.0001).

**Table 3:** Distribution of CS by different subgroups of TGCS. p-value < 0.0001; N = total number of CS in each group of TGCS; N1 = contribution of each group to total CS (%) = N/total CS×100; N2 = contribution of each group to total birth (%) =N/total deliveries×100. CS: cesarean section TGCS: Robson’s Ten Group Classification System

Robson’s group	N	N1	N2
1	126	15.51%	5.92%
2	214	26.35%	10.05%
3	24	2.95%	1.13%
4	26	3.20%	1.22%
5	284	34.97%	13.34%
6	34	4.18%	1.59%
7	22	2.71%	1.03%
8	12	1.47%	0.56%
9	12	1.47%	0.56%
10	58	7.14%	2.72%
Total CS	812		
Total deliveries	2,128		
Overall CS rate	38.16%		

**Figure 2:**
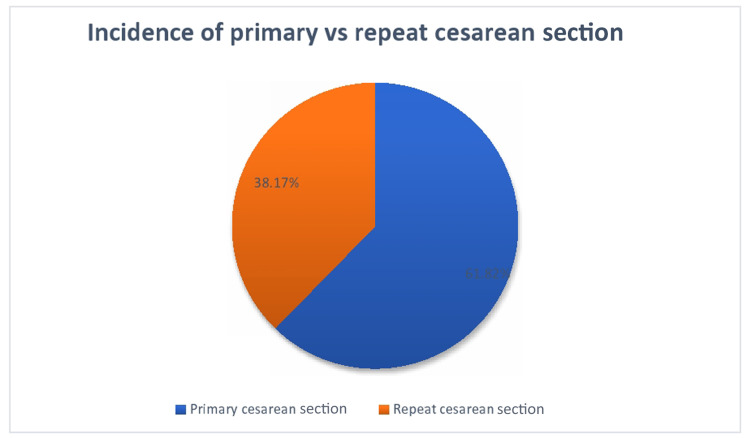
Incidence of primary versus repeat cesarean section.

Out of the total 812 cesarean deliveries, the incidence of primary CS (groups 1, 2, 3, 4, 6, 7, 8, 9, and 10) was 61.82%, while the incidence of repeat CS (group 5, 7, 8, 9, and 10) was 38.17% (Figure 2).

As shown in Table 4, the main indications for CS in group 5 of the TGCS were refusal for vaginal birth after cesarean (VBAC) (48.94%), followed by patients not suitable for VBAC (22.88%) due to various contraindications of VBAC (scar tenderness, short inter-conceptional period, cephalopelvic disproportion, severe preeclampsia, and eclampsia and cardiac disorders New York Heart Association 3/4). Failed VBAC was observed in 8.45% of patients, while the remaining patients underwent CS due to absolute indications.

**Table 4:** Indications of CS in group 5 of TGCS. VBAC: vaginal birth after cesarean; CS: cesarean section TGCS: Robson’s Ten Group Classification System

Indications of CS	Number	Percentage
Previous 1 CS not suitable for VBAC	65	22.88%
Previous 1 CS not willing for VBAC	139	48.94%
Previous 1 CS with failed VBAC	24	8.45%
Previous 2 CS	46	16.19%
Previous 3 CS	6	2.11%
Previous 4 CS	2	0.70%
Rupture uterus	2	0.70%
Total	284	100%

As shown in Table 5, 54 antenatal women with a previous scar were given a trial of labor after cesarean. Successful VBAC was seen in 55.55% of patients, while 44.44% of patients underwent emergency repeat CS due to various indications (impending scar rupture, fetal distress, non-progression of labor, and second stage arrest).

**Table 5:** Pregnancy outcomes in the VBAC group. VBAC: vaginal birth after cesarean

	Number	Percentage
Patient willing and allowed for VBAC	54	100%
Successful VBAC	30	55.55%
Failed VBAC	24	44.44%

**Figure 3:**
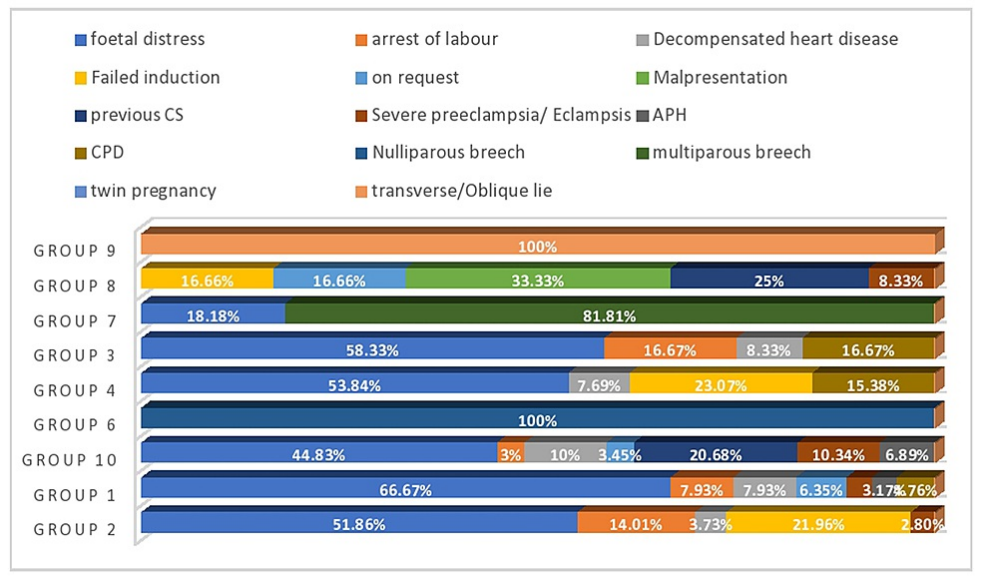
Indications of CS among other major/minor contributor groups of TGCS. CS: cesarean section; CPD: cephalopelvic disproportion; APH: antepartum hemorrhage; TGCS: Robson’s Ten Group Classification System

Figure 3 shows the main indications of CS among other major (groups 2, 1, and 10) and minor contributors (groups 6, 4, 3, 7, 8, and 9).

## Discussion

**Figure 4:**
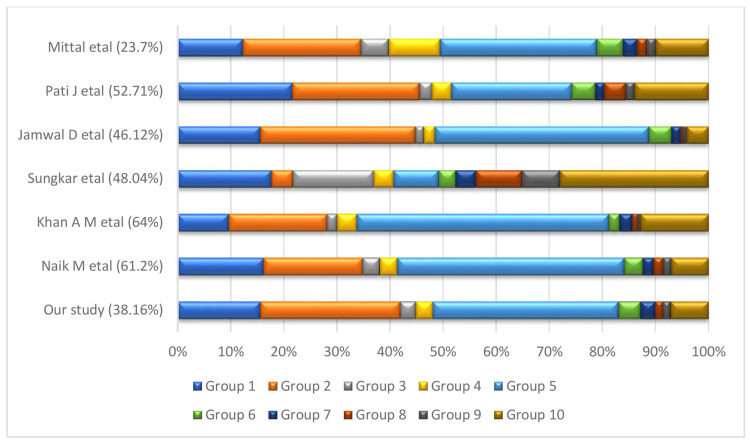
Studies showing the overall CS rate and the contribution of different TGCS groups to the overall CS rate. CS: cesarean section; TGCS: Robson’s Ten Group Classification System

In this study, the overall CS rate was 38.16% which is much higher than that proposed by the WHO (10-15%) [1]. Cesarean procedures performed in the absence of a clinical justification do not reduce maternal or infant death rates if performed at a rate higher than 10-15% [10]. Robson’s groups 5, 2, 1, and 10 were the major contributors to the overall CS rate in our institution which was similar to other studies, although in a different order [11,12]. Similarly, Vogel et al. [13] analyzed the contributions of specific groups through TGCS in two WHO multi-country surveys among seven high Human Development Index (HDI) countries, eight medium HDI countries, and six low HDI countries. In all three HDI groups, Robson groups 1 and 2 followed by group 5 were the major contributor to the overall CS rate. While in the study conducted by Pati et al. (2018), group 2 was the major contributor followed by groups 1, 3, and 10. In the study by Sungkar et al. (2019), group 10 was the major contributor, followed by groups 1, 3, and 8 [14,15] (Figure 4). Because there is no standard classification system exists for indication of CS, the indications for CS, and hence, group distribution of TGCS varies among different institutions. The difference among the contributors of different institutions clearly signifies the importance of Robson’s classification, which helps in the development of center-specific strategies and goals pertaining to particular subgroups of TGCS to control the rising CS rate.

The increasing cesarean rate has now become a growing concern at the national and international levels. The current CS rate in India is 17.2% (NFHS-4), which varies in different states, being higher in several southern states [16]. Although the state population-based CS rate of Bihar is one of the lowest in India (6.2%, NFHS-4) [16], the present study has shown a high CS rate in our institute (38.16%). This might be because our hospital is a tertiary care hospital with a superspeciality department where most of the cases are complicated and referred from various centers of Bihar.

**Figure 5:**
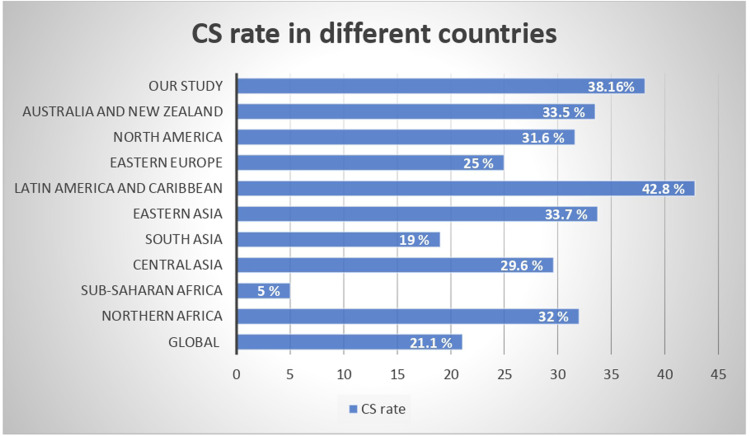
CS rates in different countries as per the latest survey (United Nations geographical grouping, 2018). CS: cesarean section

According to the United Nations geographical grouping report [17] (Figure 5), the CS rate ranges between 5% and 42.8% in different countries. Reasons for high CS rates vary widely between and within countries. This includes institution-specific policies and financing, different obstetrical risk factors and population demographics, discrepancies in a woman’s access to CS, and quality of healthcare.

The incidence of primary CS was more than repeat CS in our institute. The main indications of CS among the primary group were fetal distress, failed induction, arrest of labor, and malpresentation. Variations were noted for the indications of CS among different studies (Table 6) [18-23].

**Table 6:** Studies showing indications for CS. CS: cesarean section

Indications	Present study	Tura et al. [18]	Das et al. [19]	Maskey et al. [20]	Chavda et al. [21]	Nelson et al. [22]	Kose et al. [23]
Fetal distress	31.15%	24.3%	10.97%	28%	0.90%	18.5%	14.09%
Previous CS	36.45%	16.9%	29.96%	18%	39.90%	17%	35.72%
Arrest of labor	5.66%	9.8%	13,93%	12%	4.8%	44%	7.93%
Decompensated heart disease	3.45%	-	-	-	-	-	-
Failed induction	6.77%	-	5.21%	-	7.3%	-	12.93%
Multiple pregnancies	1.47%	-	-	-	-	-	1.09%
Malpresentation	7.88%	10.91%	6.08%	7%	18.6%	10.5%	4.44%
On request	2.7%	-	-	-	-	-	-
Severe preeclampsia/Eclampsia	3%	3.4%	4.87%	4%	-	1.5%	7.18%
Antepartum hemorrhage	0.98%	10.1%	-	-	-	4.5%	-
Cephalopelvic disproportion	1.72%	20.8%	11.84%	6.5%	19.10%	-	1.71%

Due to the significant association with overall CS rate, special attention and analysis are needed among groups 5, 2, 1, 10, and primary group, which will also help in reducing CS rate among other TGCS groups.

Previous single CS contributed to 80% of CS in group 5 of TGCS. Refusal for VBAC was the most common indication, followed by unsuitable candidates for VBAC. Reasons for refusal of VBAC included the fear that prior stitches might open up, unable to tolerate labor pain, unwilling to accept prolonged induction in case of poor Bishop’s score, and believing elective repeat cesarean delivery (ERCD) to be a safer mode of delivery, especially in patients with a previous bad obstetrical history.

VBAC is associated with decreased maternal morbidity and a decreased risk of complications in future pregnancies, as well as a decrease in the overall CS rate. Hence, for promoting VBAC, the Royal College of Obstetricians and Gynaecologists [24] recommends the routine use of VBAC checklists during antenatal counseling as they would ensure informed consent and shared decision-making in women undergoing VBAC. Women should be properly counseled regarding the benefits of VBAC as ERCD is associated with a small increased risk of placenta previa and/or accreta in future pregnancies, as well as pelvic adhesions complicating any future abdominopelvic surgery. In our study, the various reasons for unsuitable candidates for VBAC were patients with an ultrasound-documented thin scar, scar tenderness, short inter-conceptional period, cephalopelvic disproportion, severe preeclampsia and eclampsia, and cardiac disorders NYHA 3/4.

Studies have demonstrated an inverse relationship between scar thickness and the risk of scar dehiscence/rupture. However, to date, no ideal cut-off value of scar thickness has been found to be associated with successful VBAC due to the heterogenicity of the methods used to measure the lower uterine segment. According to a meta-analysis [25], a myometrial thickness (the minimum thickness overlying the amniotic cavity at the level of the uterine scar) cut-off of 2.1-4.0 mm provides a strong negative predictive value for the occurrence of a uterine defect during VBAC, whereas a myometrial thickness cut-off between 0.6 and 2.0 mm provides a strong positive predictive value for the occurrence of a uterine defect. In our institute, patients with scar thickness of ≤3 mm were not allowed for VBAC, and the decision of VBAC in patients with scar thickness of >3 mm had individual variations.

Bujold et al. [26], in their study, observed that interdelivery interval shorter than 18 months should be considered a risk factor for uterine rupture. Whereas American College of Obstetricians and Gynecologists (ACOG) [27] has advised women to avoid interpregnancy fewer than six months and should be counseled regarding the risk and benefits of repeat pregnancy sooner than 18 months. In a study by Stamilio et al. [28], an interval of fewer than six months was associated with an increased risk of uterine rupture, major morbidity, and blood transfusion twofold to threefold in VBAC candidates. In our study, women with an interpregnancy interval of <12 months were contraindicated for VBAC. Between 12 and 18 months, there was a different opinion for consideration of VBAC while women with ≥18 months interpregnancy interval were counseled for VBAC. Judicious fetal heart rate monitoring, clinical signs, and use of continuous electronic fetal monitoring can avoid the overdiagnosis of scar tenderness and reduce CS rate; moreover, twin pregnancy with first baby cephalic is also not a contraindication for VBAC.

Reduction in the rate of primary CS will further reduce the incidence of previous CS and the overall CS rate. The rate of primary CS and CS among other major contributors (groups 2, 1, and 10) can be reduced by adopting different approaches for each indication. Among the total 253 cases of fetal distress, fetal distress was seen as a reduced fetal movement with severe oligohydramnios with abnormal cardiotocography (CTG) in 7.11% of cases, severe intrauterine growth restriction (IUGR) with Doppler changes in 5.13% of cases, and meconium or blood-stained liquor in the latent phase of the first stage of labor with reactive CTG in 36.75% or with abnormal CTG trace in 50.98% of cases. For fetal distress, interobserver variations in the interpretation of CTG trace and different management approach, especially for category 2 CTG trace, requires special attention, which was the most common pattern of non-reassuring fetal heart rate for CS in our study. Currently, there is no available standard approach for the management of this condition. However, corrective measures of underlying conditions along with continuous intrapartum surveillance are required in the case of category 2 tracings. Proper training of postgraduates and senior residents for interpreting CTG trace using one of the standard guidelines, increasing the threshold for Doppler changes in IUGR fetuses, and practice of vibroacoustic stimulation test is required to reduce the CS rate for fetal distress.

In our study, among 50 cases of arrest of labor, CS was performed in 18.18% of cases in view of second stage arrest, arrest of labor in the active phase of the first stage of labor was responsible in 22.71% of cases mainly due to occipital-posterior position, and in 63.63% of cases, CS was done due to arrest of labor in the latent phase of the first stage of labor. Different thresholds for the diagnosis of non-progressive labor (NPOL) in the first stage of labor in our study require revisiting the definition of NPOL, as suggested by the ACOG and the Society of Maternal and Fetal Medicine [29]. The prolonged latent phase of the first stage of labor per se should not be an indication for CS, as most of the time, active labor is achieved with adequate and efficient uterine contraction by amniotomy with or without oxytocin administration.

Differences in opinions regarding indications of induction of labor, quantity, dosing schedule and choice of inducing agents, and duration of induction methods, especially in high-risk pregnancy, resulted in a greater number of failed induction and fetal distress, and hence, a greater number of CS in our study. Proper case selection, standard guidelines, and uniform clinical practical algorithms are needed to avoid unnecessary induction and CS.

In addition to this, proper use and interpretation of partogram, continuous labor support, external cephalic version for breech presentation, and trial of labor in twin pregnancy with the first baby in the cephalic presentation can also contribute to lowering of primary CS.

Maternal complications were seen in 52.46% of cases, and the initial assessment of neonatal status is well reflected by the good Apgar score in our study. Further studies are required to assess any short-term and long-term risks among neonates delivered by CS and whether the reduction in CS rate will result in better maternal and neonatal outcomes.

Strength of the study

This is the first study on cesarean audit using Robson’s classification at one of the tertiary care institutes of Bihar. The baseline retrospective data of our study will be used to monitor trends of CS rate over time and will form the base for future research.

Limitations of the study

The main limitation of the study was that our institute is a single tertiary care center with various superspeciality departments where most patients are complicated and referred cases; hence, our findings might be less generalizable to the entire population of Bihar. Second, because of the retrospective design of our study using existing records, some relevant information might be missing, resulting in information bias. On the other hand, Robson’s classification does not include any information regarding indications for CS and pre-existing high-risk factors in the mother or the fetus, all of which may influence CS rates.

## Conclusions

Robson’s TGCS serves as an important tool for auditing CS and can be easily implemented at institutional, state, national, and international levels for comparison of CS rates. Indications for CS among major contributors and primary groups should be analyzed regularly, and uniform and standard protocols should be used. TGCS helps in making uniform policy and strategies targeted at specific subgroups of women for optimizing CS rate. Main efforts to reduce the overall CS rate should be directed toward increasing vaginal birth after CS and reducing primary CS.

## References

[1] (2021). WHO statement on caesarean section rates. http://www.who.int/reproductivehealth/publications/maternal_perinatal_health/cs-statement/en/.

[2] (2022). NHS maternity statistics, England 2016-17. https://digital.nhs.uk/data-and-information/publications/statistical/nhs-maternity-statistics/2016-17.

[3] Wanjari SA (2014). Rising caesarean section rate: a matter of concern?. Int J Reprod Contracept Obstet Gynecol.

[4] Belachew J, Cnattingius S, Mulic-Lutvica A, Eurenius K, Axelsson O, Wikström AK (2014). Risk of retained placenta in women previously delivered by caesarean section: a population-based cohort study. BJOG.

[5] Robson MS (2001). Can we reduce the caesarean section rate?. Best Pract Res Clin Obstet Gynaecol.

[6] (2016). Best practice advice on the 10-Group Classification System for cesarean deliveries. Int J Gynaecol Obstet.

[7] Kacerauskiene J, Bartuseviciene E, Railaite DR (2017). Implementation of the Robson classification in clinical practice: Lithuania's experience. BMC Pregnancy Childbirth.

[8] Ansari A, Baqai S, Imran R (2019). An audit of caesarean section rate using modified Robson criteria at a tertiary care hospital. J Coll Physicians Surg Pak.

[9] Abubeker FA, Gashawbeza B, Gebre TM, Wondafrash M, Teklu AM, Degu D, Bekele D (2020). Analysis of cesarean section rates using Robson ten group classification system in a tertiary teaching hospital, Addis Ababa, Ethiopia: a cross-sectional study. BMC Pregnancy Childbirth.

[10] Cagan M, Tanacan A, Aydin Hakli D, Beksac MS (2021). Changing rates of the modes of delivery over the decades (1976, 1986, 1996, 2006, and 2016) based on the Robson-10 group classification system in a single tertiary health care center. J Matern Fetal Neonatal Med.

[11] Mittal P, Pandey D, Suri J, Bharti R (2020). Trend prediction for cesarean deliveries based on Robson classification system at a tertiary referral unit of North India. J Obstet Gynaecol India.

[12] Naik M, Rani S, Ratnani R (2021). Assessing cesarean section trends in a tertiary care teaching hospital using Robson’s ten group classification. Int J Health Clin Res.

[13] Vogel JP, Betrán AP, Vindevoghel N (2015). Use of the Robson classification to assess caesarean section trends in 21 countries: a secondary analysis of two WHO multicountry surveys. Lancet Glob Health.

[14] Pati T, Marandi S, Mohapatra S (2018). Analysis of caeserian section rate using Robson’s classification in a tertiary care hospital of eastern Odisha. J Med Sci Clin Res.

[15] Sungkar A, Santoso BI, Surya R, Fattah NA (2019). Classifying cesarean section using Robson classification: an Indonesian tertiary hospital survey. Maj Obs Gin.

[16] Radhakrishnan T, Vasanthakumari KP, Babu PK (2017). Increasing trend of caesarean rates in India: evidence from NFHS-4. J Med Sci Clin Res.

[17] Betran AP, Ye J, Moller AB, Souza JP, Zhang J (2021). Trends and projections of caesarean section rates: global and regional estimates. BMJ Glob Health.

[18] Tura AK, Pijpers O, de Man M, Cleveringa M, Koopmans I, Gure T, Stekelenburg J (2018). Analysis of caesarean sections using Robson 10-group classification system in a university hospital in eastern Ethiopia: a cross-sectional study. BMJ Open.

[19] Das RK, Subudhi KT, Mohanty RK (2018). The rate and indication of caesarean section in a tertiary care teaching hospital eastern India. Int J Contemp Pediatr.

[20] Maskey S, Bajracharya M, Bhandari S (2019). Prevalence of cesarean section and its indications in a tertiary care hospital. JNMA J Nepal Med Assoc.

[21] Chavda D, Goswam K, Dudhrejiya K (2017). A cross sectional study of 1000 lower segment cesarean section in obstetrics and gynecology department of P. D. U Medical College, Rajkot, Gujarat, India. Int J Reprod Contracept Obstet Gynecol.

[22] Nelson JP (2017). Indications and appropriateness of caesarean sections performed in a tertiary referral centre in Uganda: a retrospective descriptive study. Pan Afr Med J.

[23] Kose V, Sadhvi K (2020). Study of caesarean section at tertiary care center: a retrospective study. Int J Reprod Contracept Obstet Gynecol.

[24] Royal college of Obstetricians and Gynecologists (2022). Royal College of Obstetricians and Gynecologists. Birth after previous caesarean birth (green-top guideline No. 45). RCOG.

[25] Kok N, Wiersma IC, Opmeer BC, de Graaf IM, Mol BW, Pajkrt E (2013). Sonographic measurement of lower uterine segment thickness to predict uterine rupture during a trial of labor in women with previous Cesarean section: a meta-analysis. Ultrasound Obstet Gynecol.

[26] Bujold E, Gauthier RJ (2010). Risk of uterine rupture associated with an interdelivery interval between 18 and 24 months. Obstet Gynecol.

[27] (2019). Obstetric care consensus no. 8: interpregnancy care. Obstet Gynecol.

[28] Stamilio DM, DeFranco E, Paré E (2007). Short interpregnancy interval: risk of uterine rupture and complications of vaginal birth after cesarean delivery. Obstet Gynecol.

[29] (2022). Obstetric care consensus no. 1: safe prevention of the primary cesarean delivery. Obstet Gynecol.

